# Trustworthiness, Not Trust: How Systemic Racism Impacts COVID-19 Vaccine Receipt

**DOI:** 10.1089/heq.2022.0145

**Published:** 2023-07-05

**Authors:** Monique Jindal, Miao Jenny Hua, Madison Hartstein, Molly Martin

**Affiliations:** ^1^ Department of Medicine, University of Illinois Chicago, Chicago, Illinois, USA.; ^2^ Department of Preventive Medicine, Northwestern University Feinberg School of Medicine & Cook County Health, Chicago, Illinois, USA.; ^3^ Department of Medical Social Sciences, Northwestern University Feinberg School of Medicine, Chicago, Illinois, USA.; ^4^ Department of Pediatrics, University of Illinois Chicago, Chicago, Illinois, USA.

**Keywords:** trust, trustworthiness, racism, COVID

## Abstract

To illuminate the forces of structural racism influencing COVID-19 vaccine receipt, we developed a conceptual model that recontextualizes trust and presents potential pathways to address structural racism. Our model emerged from Chicagoland CEAL, a partnership of community and academic experts collaborating to encourage COVID-19 vaccine uptake for communities of color. We concluded that systemic factors influenced by racism contribute to an overall lack of trustworthiness in vaccine-affiliated institutions. We highlight the need to recenter discussions of COVID-19 vaccination on our system's trustworthiness rather than mistrust and suggest using the model to test pathways to close racial gaps in COVID-19 vaccination.

“It's a shame they don't *trust* us,” mumbled the resident as she left the precepting room and hurried to her next patient. Trust—a word often used to exonerate the medical community and our country from hundreds of years of systemic oppression—has become a common trope in COVID-19 pandemic discourse. Trust, however, is a scapegoat that leads us to an all too familiar place, where we uphold, through action and inaction, systems that do not value Black lives. Was it the moment in the examination room when the resident offered the COVID vaccine that we should focus on, or rather the hundreds of years that have led to that moment and that patient's choice?

Recognizing 400 years of slavery, Jim Crow laws, domestic terrorism, medical abuse, mass incarceration, achievement gaps, and racial health inequities in nearly every disease area, we must go beyond an emphasis on individual trust toward a coordinated commitment to Black lives demonstrated through societal actions, investments, and policies.

Fixating on trust in isolation is a form of structural racism. Structural racism refers to the totality of ways in which societies foster racial discrimination through mutually reinforcing systems such as housing, education, employment, earnings, benefits, credit, media, health care, and criminal justice.^1^ Structural racism has increasingly been recognized as a significant contributor to COVID-19 racial inequities. Across the United States, Black communities have higher case rates, more deaths, and lower vaccination rates compared with their White counterparts.^2^ Furthermore, recent research demonstrates that Black Americans who desire the vaccine are still less likely to receive it compared with the other racial groups,^3^ highlighting the influence of structural racism on vaccination rates that extends beyond individual levels of trust.^4^

Yet, much of the discourse around COVID-19 vaccines focuses on correlating individual hesitancy and mistrust with low levels of vaccine uptake. This occurs whenever a positive correlation between survey responses about vaccine intent and trust in vaccine-adjacent institutions is interpreted as signifying that trust is the most important factor in determining individual vaccine receipt and population-level vaccination rates. Pervasive in both health research literature and the popular media, such assumptions fail to interrogate the conceptual nuances of trust and often confound trust itself with the consequences of trust. Sociology scholars caution this focus on the functional properties of trust including the view that trust is simply synonymous with favorable expectations of individuals' and institutions' actions or intentions and that trust alone induces behavior.^5^

Such a focus not only neglects the complex relational process of how trust is produced but also obscures the role of oppression in making institutions of vaccine production, promotion, and dissemination objectively *untrustworthy* to the vulnerable social groups that these same institutions are now eager to study and reach.^6^

Shifting away from trust as an abstract concept or moral imperative toward *the idea of trustworthiness* underscores the social and structural inequities often perpetuated by institutions of vaccine dissemination from which individual perceptions of trust emerge. In defining “trustworthiness,” we take cues from existing models of structural determinants of health that have informed public health and epidemiological research on structural racism yet seem curiously missing from studies that depend on operationalizing the ideas of “mistrust” and “vaccine hesitancy.”^7^ Most insidiously, mistrust and vaccine hesitancy are reduced to misperceptions stemming from ignorance, ultimately erasing the *objective* conditions of structural racism, to frame vaccination and its consequences as first and foremost an individual choice.

This uncritical approach toward vaccine hesitancy as the absence of trust neglects that trust places an individual in a position of vulnerability and misses an opportunity to bring clarity onto a complex relationship between individual trust at any particular moment and the more durable conditions of institutional trustworthiness.^8^

To recenter researchers on structural rather than individual factors influencing COVID-19 vaccine receipt, we have three major recommendations. First, we recommend elucidating how trust is conceptualized when it bears an explanatory burden for vaccine-related behaviors. This can involve referencing conceptual models that exist in the public health and social sciences literature and creating causal diagrams that embed trust in relation to other structural factors. A large body of research suggests that trust is not a transparent concept that can be easily and consistently measured; it depends on social frames of interpretation and affect, even when more rationalist bases of trust are readily available.^5,8^ Second, we suggest using a hermeneutic method to studying the motivations of vaccination, such as through semistructured interviews and ethnographic research, which may bring much-needed nuance to the discourses around trust.

Lastly, we recommend focusing research efforts on the complex relationship and important distinction between individual perceptions of trust and the objective trustworthiness of a system. According to social scientists, trustworthiness includes both commitment and competency or aptitude.^8^ If we view trustworthiness as objective, we can consider what is expected of an institution and then measure whether the institution acts to support those expectations. This recommendation takes into account that mistrust is often a perfectly rational response and that an institution's trustworthiness must be developed from the objective experiences of the communities that it serves.

We have developed a conceptual model of trustworthiness by highlighting systemic components of structural racism. Some of these components have been the focus of innovative and bold approaches to COVID-19 vaccination disparities in Chicago, one of the most segregated cities in the nation. This article describes our model and how it will be used for a critical analysis of COVID-19 vaccination efforts in Chicago through the lens of structural racism.

Our model emerged from the Chicagoland CEAL Program (CCP, OT2HL161610), an NIH-funded partnership of community and academic experts collaborating to encourage COVID-19 testing and vaccine uptake for communities of color in the Chicago Region. We were informed by expert opinions from the CCP leadership team, a needs assessment with community partners, and a literature review of COVID-19 and structural racism. The primary CCP team includes five academic health centers, one community-engaged research institute, and one community advocacy organization. Led by the seven coprincipal investigators from these organizations, the CCP Steering Committee, which also includes coinvestigators, staff, community health workers, and local health leaders (*N*=33), has met every 2–4 weeks since May 2021. The CCP identified 186 community-serving COVID-19 response programs in the summer of 2021.

We conducted interviews from July to November 2021 with leadership from 35 high-priority programs with a large reach and established partnerships within Black and Latinx communities. These interviews explored racism and various programming challenges. Themes from these interviews and from published literature were discussed by the CCP Steering Committee and in a smaller working group, leading to a draft model. The model was then presented to the CCP Steering Committee again for feedback and finalized.

Figure 1 demonstrates the multilevel, mutually reinforcing factors that influence COVID-19 vaccine receipt using the socioecological model. While an individual exercises autonomy regarding what/who they trust, structural and systemic factors upstream may often overdetermine their decision. The factors contributing to structural racism at the policy, community, organization, and interpersonal levels have been well described by others. We hypothesize that these factors translate into situations of chronic social disinvestment, limited access to the vaccine, and lack of trusted messengers—contributing to an *overall lack of trustworthiness* in vaccine-affiliated institutions. For the individual, our model uses an integrated behavior model to highlight the impact of structural racism through trustworthiness on individual agency, attitudes, and norms that shape behavior.

**FIG. 1.**
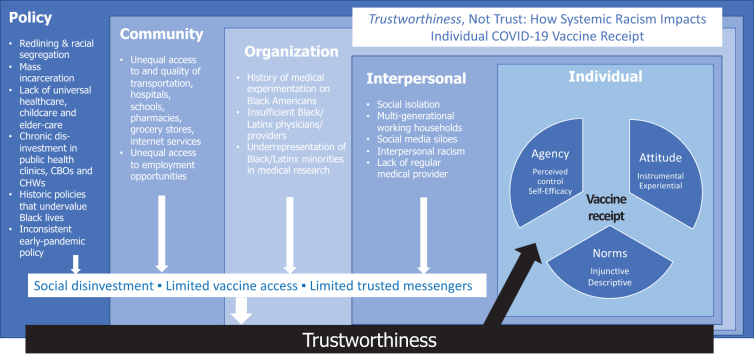
Trustworthiness and COVID-19 vaccine receipt, an adaptation of the socioecological model and integrated behavioral model.

A demonstrative example of the interconnectedness of structural racism, trustworthiness, and vaccine receipt is residential segregation. Residential segregation, or the physical separation of racial groups by systematically enforced residence, yields unequal access to resources such as transportation, high-quality hospitals/pharmacies, internet, education, and employment opportunities. Limited transportation options diminish access to drive-through vaccine services and vaccination sites predominantly located outside of segregated neighborhoods. Lack of high-quality hospitals/pharmacies limits access to vaccines themselves while poor internet services impede use of online vaccine appointment portals. Lastly, limited education and employment opportunities can impact paid time off to obtain the vaccine, access to reliable information regarding the vaccine, insurance coverage facilitating relationships with a regular medical provider, and the pipeline of potential minoritized health care professionals who may serve as trusted messengers.

These accumulated structural mistreatments, both historical and contemporary, accrue to overdetermine individual perceptions and behavior. As a community organizer said in an interview: “there is a history of medical mistreatment…right… going back generations…to slavery and even post slavery days. Folks just cannot put their trust in a system that historically has disenfranchised them and mistreated them and, in some cases, killed them.” However, this goes beyond history, another organizer stated: “…[while] there is reference to concerns because of the Tuskegee and I think that's true … but I think it's actually a more immediate and present experience… They don't need to point back to that experiment. They can point to their aunt or themselves …I wasn't taken seriously [*by the doctor*] … My aunt said do this. I did it. Hey, I got better so you've got nothing for me…”

These reflections not only highlight the complex interplay between perceptions of trust and the objective reality of structural racism and community disinvestment, but also illuminate the critical need for patient voice. Future research can center institutions and health care systems as the responsible party in becoming trustworthy. This does not mean a push to neglect micro- or individual-level factors, but rather emphasizes the importance of measuring both individual and structural factors and acknowledging their relationship to begin to understand what components are necessary to become a trustworthy institution and how these components might be leveraged to improve process outcomes such as vaccine receipt.

Structural forces have colluded to limit the effectiveness of short-term equity-oriented interventions to improve vaccine receipt. In Chicago, for instance, the Black-White disparity in vaccine receipt remains stark despite city-led mitigation measures targeting racial inequity.^9^ These efforts include a program (Protect Chicago Plus) to focus vaccination efforts in neighborhoods with the highest social vulnerability index and a larger effort to deploy hyperlocal strategies for COVID-19 vaccination throughout the entire city (Healthy Chicago Equity Zones). The longer term effectiveness of these programs to close the vaccination gap between Black and White Chicagoans is yet to be demonstrated. Our model provides a framework for this analysis and its interpretation.

Overall, our model highlights the urgent need to center discussions of COVID-19 vaccination on system trustworthiness and provides a framework for testing potential pathways to close the stark racial gaps in COVID-19 vaccine receipt. Our next steps are to use the model to test the interconnectedness of the proposed pathways to COVID-19 vaccination, in an effort to reduce the incessant exhortations for individuals to *trust* and instead recenter on our obligation to make our systems more *trustworthy*.

## Abbreviation Used

CCP: Chicagoland CEAL Program
